# Injuries in Elite Men’s Rugby Union: An Updated (2012–2020) Meta-Analysis of 11,620 Match and Training Injuries

**DOI:** 10.1007/s40279-021-01603-w

**Published:** 2021-12-02

**Authors:** Sean Williams, Charli Robertson, Lindsay Starling, Carly McKay, Stephen West, James Brown, Keith Stokes

**Affiliations:** 1 Centre for Health and Injury and Illness Prevention in Sport, Department for Health, University of Bath, Bath, BA2 7AY UK; 2 Faculty of Kinesiology, Sport Injury Prevention Research Centre, University of Calgary, Calgary, Canada; 3 O’Brien Institute for Public Health, University of Calgary, Calgary, Canada; 4 Division of Orthopaedic Surgery, Department of Surgical Sciences, Faculty of Medicine and Health Sciences, Institute of Sport and Exercise Medicine, Stellenbosch University, Stellenbosch, South Africa; 5 Rugby Football Union, Twickenham, UK; 6 IOC Research Centre, Pretoria, South Africa

## Abstract

**Background:**

The most recent meta-analytic review of injuries in elite senior men’s Rugby Union was published in 2013. The demands of the game at the elite level are continually changing alongside law amendments and developments in player preparation. As such, an updated meta-analysis of injury data in this setting is necessary.

**Objective:**

To meta-analyse time-loss injury data in elite senior men’s Rugby Union between 2012 and 2020.

**Methods:**

Electronic databases were searched using the keywords ‘rugby’ and ‘inj*’. Nineteen studies met the inclusion criteria. Injury incidence rate data were modelled using a mixed-effects Poisson regression model. Days missed data were modelled using a general linear mixed model.

**Results:**

The included data encompassed a total of 8819 match injuries and 2801 training injuries. The overall incidence rate of injuries in matches was 91 per 1000 h (95% confidence interval (CI) 77–106). The estimated mean days missed per match injury was 27 days (95% CI 23–32). The overall incidence rate of match concussions was 12 per 1000 h (95% CI 9–15). The overall incidence rate of training injuries was 2.8 per 1000 h (95% CI 1.9–4.0). Playing level was not a significant effect modifier for any outcome.

**Conclusions:**

The injury incidence rate and mean days missed per injury in the present meta-analysis were higher, but statistically equivalent to, the 2013 meta-analysis (81 per 1000 h and 20 days, respectively). The injury incidence rate for match injuries in elite senior men’s Rugby Union is high in comparison to most team sports, though the training injury incidence rate compares favourably. The tackle event and concussion injuries should continue to be the focus of future preventative efforts.

**Supplementary Information:**

The online version contains supplementary material available at 10.1007/s40279-021-01603-w.

## Key Points

**Table:** 

The incidence rate for match injuries in elite senior men’s Rugby Union is high in comparison to most team sports (91 per 1000 h), though the training injury incidence rate compares favourably (2.8 per 1000 h).
The mean days missed per match injury was 27 days.
Playing level was not a significant effect modifier for any injury outcome.
The tackle event and concussion injuries should continue to be the focus of future preventative efforts.

## Introduction

Rugby Union is amongst the most played and watched sports in the world, with approximately 9.6 million registered players in over 123 countries [1]. The game is physically demanding, with bouts of walking, jogging and running, interspersed with sprinting, static exertions and contact events [2]. Moreover, the demands of the game at the elite level are continually changing alongside law amendments [3] and developments in match analysis, equipment, technology and player training [4]. The most recent meta-analytic review of injuries in elite senior men’s Rugby Union was published in 2013 [5]. This meta-analysis confirmed that match injury incidence rates in elite senior men’s Rugby Union can be considered high in comparison to other team sports (overall injury incidence rate: 81 per 1000 h), but similar to other collision-based sports [5]. It was recommended that injury prevention efforts should target lower-limb injury prevention strategies and technique during contact to reduce the burden of injury in the sport.

Since the publication of the most recent meta-analysis in 2013, efforts to reduce injury risk in elite senior men’s Rugby Union have been undertaken [6, 7], primarily in response to rapidly increasing rates of concussion injuries [8]. These increases are thought to be due, in part, to the introduction of processes to better identify and manage head impact events during matches [9] as well as increased awareness and education around concussions. However, a real change in concussion risk resulting from changes to the demands of the elite game is also likely. Concern about potential long-term problems associated with concussion and/or multiple head impacts (i.e., neurodegenerative diseases) is recognised by medical and lay populations [10], and is a prominent issue in elite Rugby Union. Given the changes that have occurred within elite Rugby Union since 2013, and the volume of data published from new and existing injury surveillance projects, there is a clear need for an updated review and meta-analysis of injuries in this population. An updated meta-analysis will provide the most precise and up-to-date estimates of injury risk to best inform future preventative strategies in elite senior men’s Rugby Union.

## Objective

The objective of this review is to meta-analyse the epidemiological data of time-loss injuries in elite senior men’s Rugby Union between 2012 and 2020, with specific reference to: match injury incidence rates; days missed for match injuries; match concussion incidence rates; and training injury incidence rates. The effect of playing level and position as a moderating factor are also explored. In addition, the proportion of match injuries as a function of injury location and match event are summarised.

## Methods

Guidelines for reporting meta-analysis of observational studies in epidemiology (MOOSE guidelines) [11] were followed, including specifications for reporting background, search strategy, methods, results, discussion and conclusion. Details of the protocol for this systematic review were registered on PROSPERO and can be accessed at: www.crd.york.ac.uk/PROSPERO/display_record.asp?ID=CRD42020200627.

### Literature Search

Web of Science, PubMed and Google Scholar databases were searched (by SWi) from September 2012 through October 2020 using the keywords “rugby” and “inj*”. Furthermore, the reference lists of included studies and relevant ‘grey literature’ (e.g., conference proceedings and online annual injury surveillance reports) were searched to identify additional articles. Inclusion criteria for retrieved studies were: (1) prospective cohort studies; (2) study population comprising 15-a-side senior elite male Rugby Union teams; (3) use of a 24-h time-loss injury definition [12]; (4) full-text version available in English; (5) data pertaining to 2012 onwards; and (6) reports injury incidence rates for one or more of the following epidemiological data: (1) match or training injuries; (2) match concussion injuries; (3) match injuries for forwards and backs; (4) location of match injuries; (5) match event associated with injury. In addition, the mean/median days missed per match injury were extracted. Literature was excluded if appropriate injury incidence rate and either injury count or exposure time data were not reported or could not be obtained from the corresponding author. ‘Elite’ was defined as playing at international level, the top two leagues in a tier-one nation, or the top league in a tier-two nation. Duplicate records were identified and removed. Titles and abstracts of the remaining studies were assessed for relevance by SWi, CR and LS, with non-relevant articles being discarded. All articles were screened by at least two reviewers. Full-text versions of the outstanding articles were then retrieved and evaluated against the inclusion criteria by two independent review groups (SWi and CR, Cohen's kappa index value = 88.9%; LS and SWe; Cohen's kappa index value = 93.6%), with any conflicts resolved by KS (*n* = 16).

### Assessment of Reporting Quality and Risk of Bias

The reporting quality of included studies was evaluated by two independent reviewers (SWi and CR) using the ‘Strengthening the Reporting of Observational Studies in Epidemiology’ (STROBE) Sports Injury and Illness Surveillance (-SIIS) statement [13]. This 23-item checklist provides guidance on the reporting of observational studies on injury and illness in sports, but was not intended as a direct assessment of study quality. Discrepancies were resolved via discussion. The risk of small study bias was examined visually through funnel plots [available in the Online Supplementary Material (OSM)].

### Data Extraction

For studies meeting the inclusion criteria, information pertaining to the level of play, study setting, surveillance period, number of injury events, mean/median days missed per injury, and exposure time was extracted. Where only two of injury count, injury incidence rate or exposure data were provided, the missing component was calculated using the available data (e.g., where injury count and injury incidence rate data were provided, the exposure time was calculated as: ‘injury count/injury incidence rate × 1000’). Note, this approach may result in small rounding errors, but these have a negligible impact on the reported outcomes. Where relevant, multiple rows of data were extracted for each study to allow for the various combinations of counts and exposures required for each fixed effect (i.e., match injuries, training injuries, concussion injuries, body location, match event, and playing position (forwards/backs)). Data pertaining to seasons prior to 2012 were not extracted [8, 14, 15].

World Rugby organises its member unions into six tiers according to playing strength and potential [16]: Tier-one teams participate in the Six Nations Championship (England, France, Ireland, Italy, Scotland and Wales) or The Rugby Championship (Argentina, Australia, New Zealand and South Africa) while tier two currently consists of Canada, USA, Uruguay, Georgia, Portugal, Romania, Russia, Spain, Namibia, Fiji, Samoa and Tonga. For ‘level of play’, club teams were considered to be ‘level one’ if they played in the highest league within a tier-one ranked nation, and ‘level two’ if they played in the second tier of a tier-one ranked nation, or in the highest league within a tier-two ranked nation [5]. Data from international teams and tournaments (e.g., Rugby World Cups) were categorised as ‘international’. International Under-20 rugby tournaments [14] were categorised as ‘level two’. Where required, authors were contacted to obtain any additional data that were not available in the full text versions; this was necessary for two of the included studies. Where multiple studies captured the same study period for a given team, preference was given to the study with the greatest overall exposure and/or via liaison with the original authors.

### Analysis and Interpretation of Results

Statistical modelling was performed using the *metafor* package [17] in *R* (version 4.0.2, R Foundation for Statistical Computing, Vienna, Austria). Incidence rate data were modelled using a mixed effects Poisson regression model. The response variable was the number of observed injuries, offset by the log of the number of exposure hours. Injury location and match-event incidence-rate data were summarised as a proportion of all injuries in the given individual study, and then analysed via a random-effects model with raw proportions [17]. Days missed data were modelled using a general linear mixed model [17]. Between-study heterogeneity was assessed with the *I*^2^ statistic, where values of 25%, 50% and 75% represented low, moderate and high levels, respectively [18]. There were high levels of heterogeneity in all injury outcomes reported, and thus a random-effects term was included to allow for residual heterogeneity among studies and to account for the correlation arising from using multiple rows of data from the same study. Comparisons between playing levels and positional groups (forwards vs. backs) were made by including these variables as fixed effects. For the analysis of playing position, total exposure time was multiplied by 0.53 and 0.47 for forwards and backs, respectively, to account for the relative playing exposure for these positional groups (i.e., eight forwards and seven backs per team). All estimates are presented with 95% confidence intervals (CIs), with significance set at an alpha level of 0.05.

## Results

**Fig. 1:**
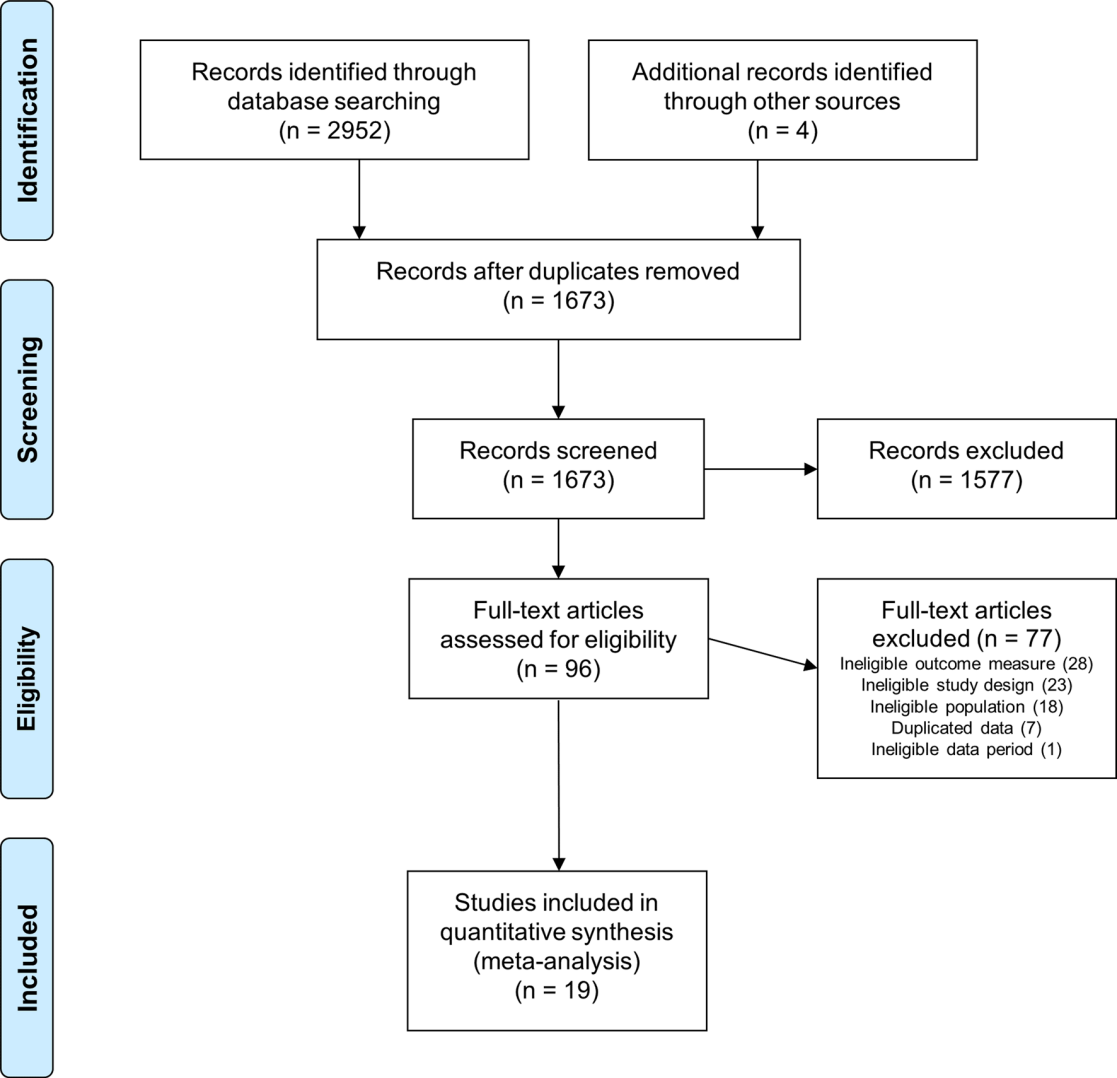
PRISMA flow diagram of the study selection process

The electronic searches returned 2952 results. After removing duplicate and non-relevant records, 96 potentially relevant studies were assessed for inclusion in this review (Fig. 1). Nineteen prospective cohort studies were included in the final analysis, encompassing a total of 8819 match injuries and 2801 training injuries. The mean ± SD reporting quality, as assessed by the 23-item STROBE-SIIS (Strengthening the Reporting of Observational studies in Epidemiology—Sports Injury and Illness Surveillance extension) checklist, was 17 ± 3 with a range of 9–21 (Table 1). Each individual rating for the STROBE-SIIS items is available in the OSM. Visual inspection of the funnel plots did not reveal any evidence of publication bias (see OSM).

**Table 1:** Study characteristics, injury data and STROBE-SIIS reporting quality of included studies

References	Setting	Level of play	Surveillance period	Activity	Injury count	Exposure time (h)	Incidence (no./1,000 h)	Mean days missed	Injury burden (days/1,000 h)	STROBE-SIIS rating [/23]
Bitchell et al. [19]	Wales	Level 1	2012–2016	Match	1086	10,960	99.1	26	2570	17
				Match (concussion)	168	10,960	15.3			
Cosgrave & Williams [20]	Ireland	Level 1	2016–2017	Match (concussion)	46	2590	17.8			18
	Ireland	International	2016–2017	Match (concussion)	5	180	27.8			
Cousins et al. [21]	England	Level 2	2017–2018	Match	125	728	171.7			12
				Training	76	12,233	6.2			
Cruz-Ferreira et al. [22]	Portugal	Level 2	2014–2015	Match	28	420	66.7	23	1501	12
Fuller et al. [23]	World Cup	International	2015	Match	173	1920	90.1	30	2685	19
				Match (concussion)	24	1920	12.5			
				Training	20	17,403	1.1			
Fuller et al. [14]^a^	World Cup	U20s (Level 2)	2012–2016	Match	273	5800	47.1	38	1720	21
				Match (concussion)	21	5800	3.6			
Fuller et al. [24]	World Cup	International	2019	Match	143	1800	79.4	29	2296	21
				Match (concussion)	22	1800	12.2			
				Training	25	16,667	1.5			
Kemp et al. [25]	England	International	2012–2019	Match	189	1695	111.5	21	2308	17
				Training	173	27,453	6.3			
Lanzetti et al. [26]	Italy	Level 2	2014–2015	Match	40	360	111.1			9
				Training	37	12,320	3.0			
Moore et al. [27]	Wales	International	2012–2014	Training	41	8737	4.7			19
				Match (concussion)	11	800	13.8			
Rafferty et al. [28]	Wales	International	2012–2016	Match	177	1000	177			20
Schwellnus et al. [29]	South Africa	Level 1	2012–2016	Match	802	8032	99.9	18	1796	20
				Match (concussion)	60	8032	7.5			
				Training	134	85,609	1.6			
Starling et al. [30]^b^	South Africa	Level 1	2014–2017	Match	502	6160	81.5			15
			2016–2017	Match				33		
			2014–2017	Match (concussion)	42	6160	6.8			
Starling et al. [31]	South Africa	Level 1	2018	Match	77	940	81.9	31		15
				Match (concussion)	14	940	14.9			
Starling et al. [32]	South Africa	Level 1	2019	Match	90	957	94	13	1222	15
				Match (concussion)	11	957	11.5			
Stokes et al. [7]^c^	England	Level 2	2019	Match	256	3600	71.1			15
				Match (concussion)	61	3600	16.9			
West et al. [15]^a^	England	Level 1	2012–2019	Match	4747	55,642	85.3	30	2602	19
				Match (concussion)	838	55,642	15.1			
West et al. [8]^a^	England	Level 1	2012–2018	Training	2245	872,823	2.6			17
Whitehouse et al. [33]	Australia	Level 1	2014	Match	111	1680	66.1	40	2630	18
				Training	50	21,459	2.3			

*STROBE-SIIS* Strengthening the Reporting of Observational studies in Epidemiology—Sports Injury and Illness Surveillance extension

^a^Data for seasons pre-2012 were not extracted

^b^Injury severity data were only captured from 2016 onwards

^c^Only control period data were extracted

### Match Injury Incidence Rate

**Fig. 2:**
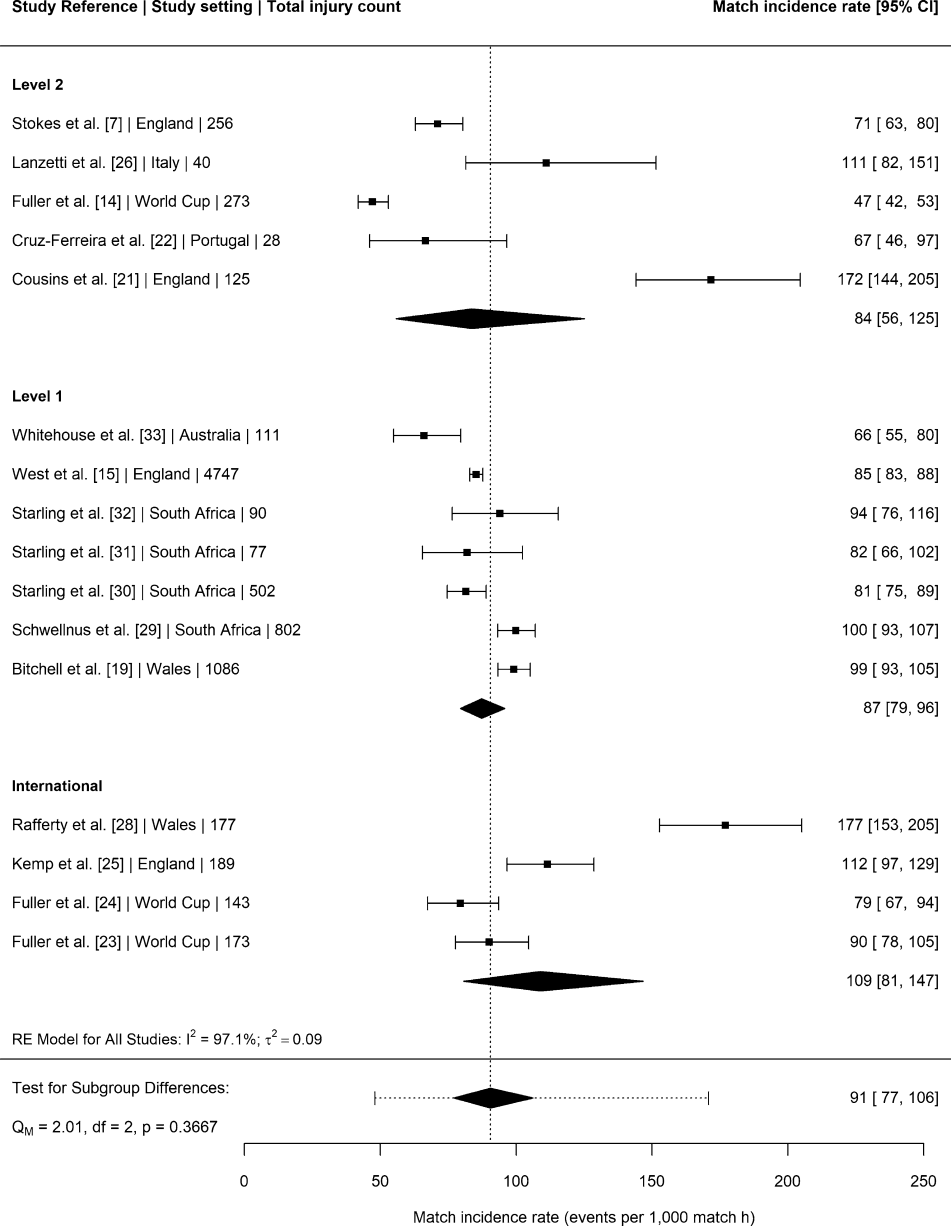
Incidence of match injuries (with 95% confidence intervals) by level of play. Study reference, study setting and total number of injury events are provided for each study. The location of the diamond represents the estimated incidence rate and the width of the diamond reflects the precision of the estimate. The dashed line represents the prediction interval, which shows the range of the true effect in 95% of study settings

Seventeen studies [7, 14, 15, 19, 21–33] provided injury surveillance data for match injuries that could be included in the meta-analysis. The 17 studies reported a total of 8819 injuries amongst elite senior male Rugby Union players exposed to 101,694 h of match time. This yielded an overall injury incidence rate of 91 per 1000 h (95% CI 77–106). Level of play was not a significant moderator of this relationship (*P* = 0.37). The mean match incidence rates per 1000 h with 95% CI were, in descending order: international: 109 (95% CI 81–147); level one: 87 (95% CI 79–96); and level two: 84 (56–125) (see Fig. 2).

### Days Missed per Injury

**Fig. 3:**
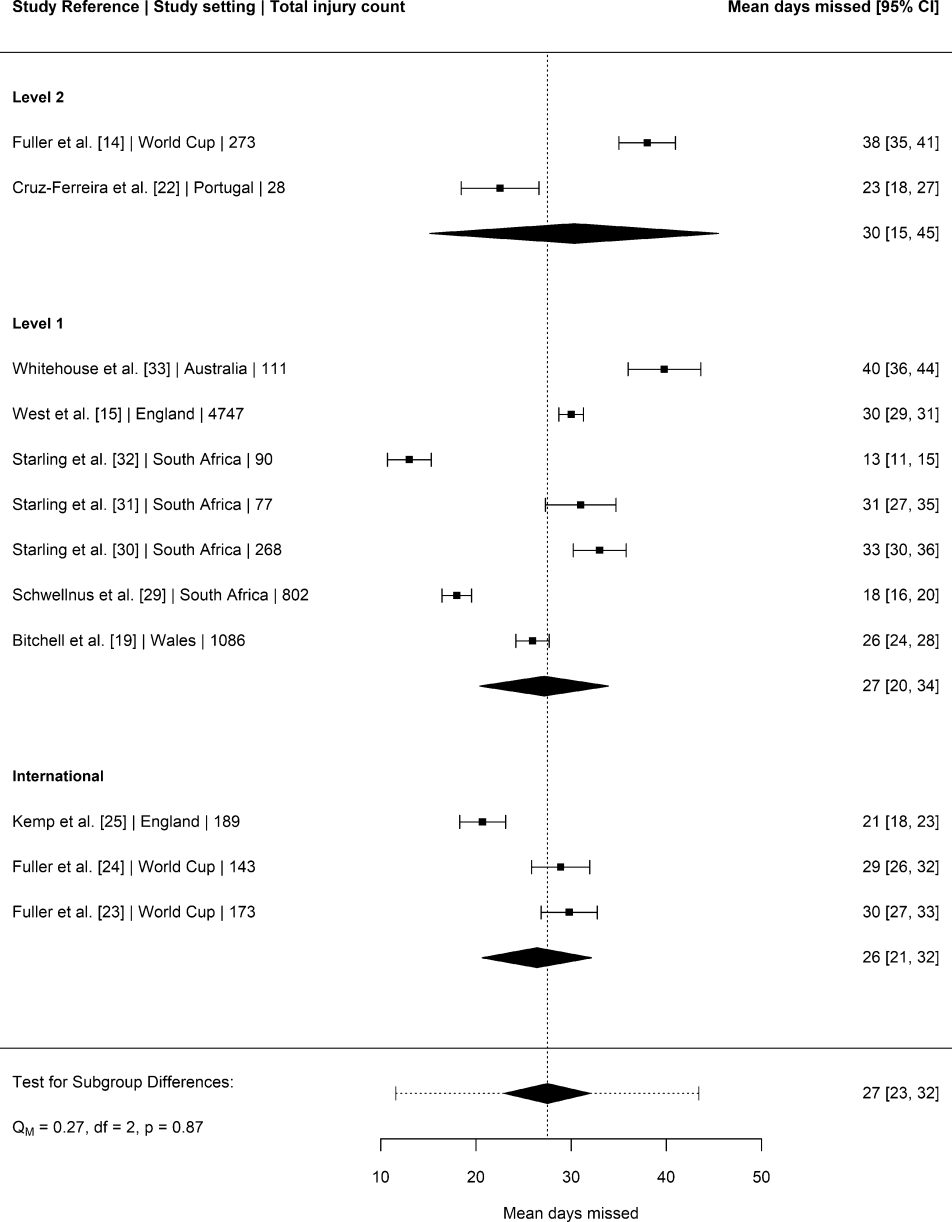
Mean days missed for match injuries (with 95% confidence intervals) by level of play. Study reference, study setting and total number of injury events are provided for each study. The location of the diamond represents the estimated mean days missed and the width of the diamond reflects the precision of the estimate. The dashed line represents the prediction interval, which shows the range of the true effect in 95% of study settings

Twelve studies [14, 15, 19, 22–25, 29–33] provided mean days missed data for match injuries that could be included in the general linear mixed model (see Fig. 3). The estimated mean days missed per match injury was 27 days (95% CI 23–32), with no significant difference across playing levels (*P* = 0.87). Nine studies [14, 15, 19, 23, 24, 28, 30–32] provided median days missed data for match injuries. The estimated median days missed per match injury was 8 days (95% CI 4–11), with no significant difference across playing levels (*P* = 0.87).

### Concussion

**Fig. 4:**
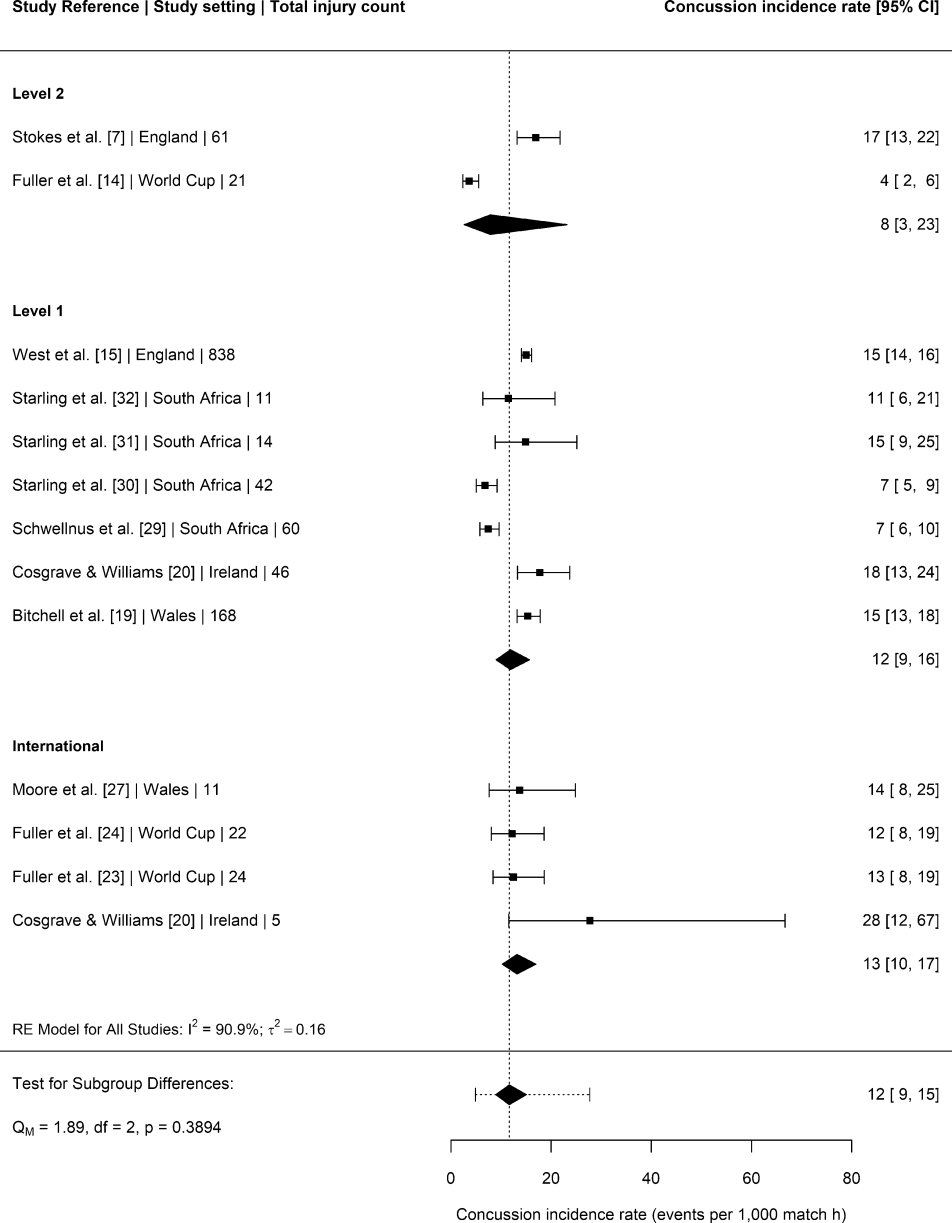
Incidence of match concussion injuries (with 95% confidence intervals) by level of play. Study reference, study setting and total number of injury events are provided for each study. The location of the diamond represents the estimated incidence rate and the width of the diamond reflects the precision of the estimate. The dashed line represents the prediction interval, which shows the range of the true effect in 95% of study settings

Twelve studies [7, 14, 15, 19, 20, 23, 24, 27, 29–32] provided match concussion incidence rate data that could be included in the meta-analysis (Fig. 4). The 12 studies encompassed a total of 1323 concussion injuries amongst elite senior male Rugby Union players exposed to 99,381 h of match time. The overall rate of match concussions was 12 per 1000 h (95% CI 9–15), with no significant moderating effect of playing level (*P* = 0.39).

### Match Injury Locations

For each injury location, a range of between four to ten studies provided data that could be included in the meta-analysis (Table 2). During matches, the head (16.7%), knee (12.9%) and shoulder (11.7%) were the most common injury locations.

**Table 2:** Match injuries as a function of injury location. Injury location incidence rate data were summarised as a proportion of all injuries in the given individual study; proportions from each study were then combined in the meta-analysis

Injury location	Number of studies	Total injury count	Meta-analysed proportion (95% CI)
Head	10	1439	16.7% (13.5–19.9)
Knee	10	1034	12.9% (12.1–13.6)
Shoulder	10	933	11.7% (9.6–13.8)
Ankle	9	312	9.3% (7.9–10.7)
Posterior thigh	8	447	6.5% (5.3–7.7)
Lower leg	10	570	6.5% (5.5–7.5)
Anterior thigh	8	338	6.0% (4.4–7.6)
Chest	6	311	4.0% (1.9–6.1)
Hip/groin	10	330	3.8% (2.6–5.1)
Wrist/hand	10	177	3.6% (2.4–4.7)
Upper back	4	28	3.1% (0.7–5.6)
Neck	9	338	2.9% (1.7–4.1)
Foot	9	84	2.4% (1.8–3.0)
Lower back	10	161	1.8% (1.5–2.2)
Elbow	7	33	1.2% (0.7–1.7)
Pelvis/sacrum	4	22	1.2% (0.2–1.9)
Upper arm	6	47	0.7% (0.5–0.9)
Abdomen	4	38	0.7% (0.5–0.9)
Forearm	6	49	0.7% (0.5–0.9)

### Match Injury Events

For each match injury event, a range of between six and nine studies provided data that could be included in the meta-analysis (Table 3). During matches, making tackles (23.0%), being tackled (22.8%), and collisions (14.2%) were the most common injury events.

**Table 3:** Match injuries as a function of match event. Match-event incidence-rate data were summarised as a proportion of all injuries in the given individual study; proportions from each study were then combined in the meta-analysis

Match event	Number of studies	Total injury count	Meta-analysed proportion (95% CI)
Tackling	9	1497	23.0% (20.7–25.2)
Tackled	9	1633	22.8% (20.7–24.9)
Collision	7	737	14.2% (10.2–18.2)
Running	9	713	10.4% (7.5–13.3)
Ruck	9	627	8.9% (6.8–11.0)
Scrum	9	257	4.3% (3.1–5.4)
Maul	5	131	2.2% (1.9–2.6)
Lineout	5	77	1.3% (1.0–1.6)
Kicking	6	30	0.6% (0.2–1.0)

### Playing Position

Eleven studies [14, 15, 19, 22–24, 29–33] that reported match-injury incidence rates for both forwards and backs were combined in the pooled analysis. The overall match-injury incidence rate was not significantly different (*P* = 0.95) between forwards (78 per 1000 h; 95% CI 66–91) and backs (76 per 1000 h; 95% CI 60–97). Nine studies [14, 15, 19, 22–24, 31–33] also provided mean days missed data for these grouped playing positions that could be included in the general linear mixed model. The mean days missed per injury was significantly higher in forwards (31 days) versus backs (27 days, meta-analysed difference = 4 days; 95% CI 3–5; *P* < 0.001).

### Training Injury Incidence Rates

**Fig. 5:**
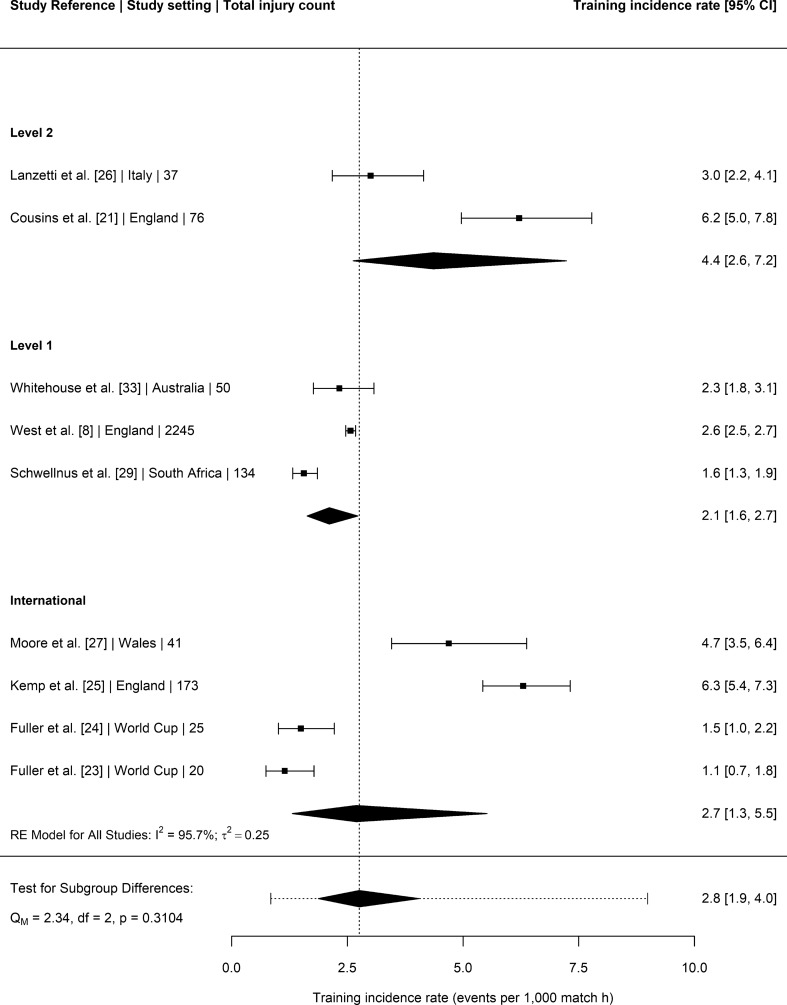
Incidence of training injuries (with 95% confidence intervals) by level of play. Study reference, study setting and total number of injury events are provided for each study. The location of the diamond represents the estimated incidence rate and the width of the diamond reflects the precision of the estimate. The dashed line represents the prediction interval, which shows the range of the true effect in 95% of study settings

Nine studies [8, 21, 23–27, 29, 33] provided injury surveillance data for training injuries that could be included in the meta-analysis (Fig. 5). The nine studies encompassed a total of 2801 injuries amongst elite senior male Rugby Union players exposed to 1,074,704 h of training time. The overall incidence of injuries in senior men’s elite rugby training was 2.8 per 1000 h (95% CI 1.9–4.0). Level of play was not a significant moderator of this relationship (*P* = 0.31).

## Discussion

The current study presents an updated (2012–2020) overview of injury data in elite senior men’s Rugby Union. This work represents the largest analysis of injuries in this setting to date, encompassing a total of 11,620 injuries. The overall match and training incidence rates in the present analysis were comparable to the 2013 meta-analysis [5]. These data confirm that the injury incidence rate for match injuries in elite senior men’s Rugby Union is amongst the highest of all team sports, though the training injury incidence rate compares favourably [34, 35]. The incidence rate for match concussion injuries is relatively high, and primary and secondary preventative efforts are a priority for this setting. The tackle accounts for the majority of match injury events and should continue to be the focus of future preventative efforts.

The overall meta-analysed match injury incidence rate was 91 per 1000 h (95% CI 77–106), with no significant moderating effect of playing level. The 2013 meta-analysis reported substantial differences between playing levels, primarily driven by a significantly lower incidence rate in the level two setting (35 per 1000 h) [5] in comparison to the current study (84 per 1000 h). These findings may reflect improved reporting of injuries in level two settings, and greater homogeneity in medical support between playing levels. The injury incidence rates in level one club (87 per 1000 h) and international (109 per 1000 h) settings in the current meta-analysis were equivalent to the values reported in the 2013 meta-analysis (89 and 123 per 1000 h, respectively) [5]. It should be noted that data relating to international teams were typically collected in a tournament setting (e.g., World Cups), which may be inherently different to matches played throughout seasonal club competitions due to differences in match scheduling, reporting practices, and greater disparities in resources and playing abilities between teams.

There were no significant differences in the mean days missed due to injury between levels of play. The mean days missed per match injury in the present meta-analysis was 7 days higher than the 2013 meta-analysis (20 vs. 27 days) [5], although this difference was not statistically significant. A recent longitudinal analysis has, however, reported significant increases in the mean days missed per injury in level one clubs over the last 16 seasons [15]. Future research should incorporate mixed method approaches (both qualitative and quantitative) to further explore the mechanism behind the increasing mean days missed per injury in this setting.

The overall rate of match concussions was 12 per 1000 h (95% CI 9–15), with no significant moderating effect of playing level. However, this meta-analysed rate does not portray the changes in concussion reporting over time. For instance, in the English Premiership concussion rates have risen from ~ 5 per 1000 h in 2011–2012 to ~ 20 per 1000 h in recent seasons [15]. The increase in concussion incidence rates is likely due to a number of factors: the introduction of processes to better identify and manage head impact events during matches [9], a lowering of the diagnostic threshold [36], increased awareness and education around concussions [36], and also a likely real change in concussion risk resulting from alterations to the demands/laws of the elite game (e.g., increased tackle frequency [3]). In many settings, concussion has emerged as the most common match injury [15], and this is supported by the head being the most common injury location in the current analysis (17% of all match injuries). The concern about potential long-term problems (e.g., neurodegenerative diseases) associated with concussion and/or multiple head impacts is recognised by medical and lay populations [10], and therefore governing bodies should continue to develop and evaluate strategies to lower the risk of concussion in elite senior men’s Rugby Union. This may include law changes [6] and limiting contact exposure in training [37].

The tackle remains the match event associated with the largest proportion of injuries, with a similar risk evident for the ball carrier and tackler (being tackled = 23%, tackling = 23%). In the previous meta-analysis, being tackled (~ 36% of all injuries) was associated with a substantially higher injury incidence rate than making tackles (~ 23% of all injuries) [5]. Potential preventative efforts related to the tackle event that are currently being trialled include more stringent sanctioning of illegal high-contact tackles and reducing the height of the tackle [7]. Future strategies may address deficiencies in tackling technique on the non-dominant side [38], and developing technical capacity to resist the effects of physical fatigue during the tackle [39]. Elsewhere, there is some evidence that the incidence of running injuries, which accounted for 10% of all match injuries, has decreased [15]. There may be further scope for risk reduction of running-related injuries through targeted injury-prevention programmes [40, 41]. The proportion of injuries associated with the scrum (4%) was lower than reported in the 2013 meta-analysis (9%) [5], which may be related to the game-wide introduction of a ‘PreBind’ technique in 2013–2014 that was shown to reduce the biomechanical loading on players during scrum engagements [42]. This demonstrates how the full injury prevention cycle can be effectively applied in elite team sports [43]. However, it should be noted that changes to the number of scrum events per match across this period may also account for this decrease in scrum-related injuries [44].

The mean days missed per injury was significantly higher in forwards versus backs (meta-analysed difference = 4 days, 95% CI 3–5), though no differences in injury incidence rates were observed. The higher mean days missed per injury in forwards may be a result of their involvement in a higher frequency and number of collisions per match [45]. In particular, forwards are involved in more tackle and ruck events than backs, which are considered amongst the highest burden events and which have increased in frequency per match over recent seasons [15]. These positional demands directly influence the assessment of activity risk within the return-to-play decision framework following an injury [46], which may also account for the observed difference in the mean days missed per injury between forwards and backs. There are likely to be position-specific differences in match injury profiles, determined by the physical and technical requirements of each position, which may be used to design more targeted injury-prevention programmes [47]. These position-specific injury profiles warrant an updated investigation, given the changes to game and positional demands that have occurred over recent seasons [3].

The overall meta-analysed training injury incidence rate in senior men’s elite Rugby Union was 2.8 per 1000 h (95% CI 1.9–4.0) with no significant moderating effect of playing level. This training injury incidence rate was equivalent to the rate reported in the 2013 meta-analysis (3 per 1000 h; 95% CI: 2–4) and compares favourably with rates reported in sports such as men’s professional football (4 per 1000 h [35]) and field hockey (4.2 per 1000 h [34]), implying that elite Rugby Union teams manage the risk associated with contact elements of training effectively. Despite the relatively low incidence rate of training injuries, they nonetheless occur in a largely controllable environment and represent a substantial proportion of all injury events (approximately one-third) [8]. Therefore, injury reduction strategies targeted at this aspect of the game have the potential to substantially reduce the overall burden of injury as well as improving career longevity of those players involved at the elite level of the game.

There may be some limitations affecting the outcomes of this meta-analysis. Injury surveillance data are reported across a range of sources, including websites, theses, conference abstracts, and stakeholder reports [48]. As such, it is possible that relevant surveillance data, both injury and exposure quantification, may have been missed. However, the extensive and systematic search strategy (including grey literature) used in the present meta-analysis is likely to have captured the vast majority of relevant data in elite senior men’s Rugby Union. Whilst all included studies used a 24-h time-loss injury definition and followed the consensus statement for injury surveillance in Rugby Union [12], methodological differences between settings (i.e., differences in who records the injury data, how data are recorded [e.g., online vs. paper-based forms), and the study setting (e.g., short tournament vs. whole-season competition)] may influence the completeness and validity of the data [48]. Since all injury-report measures are likely to have some degree of error, true ‘gold standard’ sources rarely exist, and therefore quality assessment of injury surveillance studies is difficult to undertake [48]. Injury surveillance systems in elite senior men’s Rugby Union should endeavour to assess and report the quality and completeness of their data in future publications [13, 49]. For instance, the largest study in the present meta-analysis (*n* = 4747 injuries) was overseen by a lead researcher at the host institution, who implemented a quality control process to ensure all injury details were captured on a regular basis, and undertook a validation of reported match injuries using match report cards completed by match officials [15]. Finally, these data only relate to the elite men’s game, and so are not generalisable to other Rugby Union populations.

## Conclusions

The overall match and training injury incidence rates in senior men’s elite Rugby Union were 91 per 1000 h and 2.8 per 1000 h, respectively. Playing level did not significantly moderate any of the outcome measures. These data confirm that the injury incidence rate for match injuries in elite senior men’s Rugby Union, and the rate of concussion/head injuries in particular, is amongst the highest of all team sports. The tackle accounts for the majority of these match injury events. Whilst the training injury incidence rate compared favourably with other team sports, injury-reduction strategies targeted at this aspect of the game have the potential to substantially reduce the overall burden of injury and improve career longevity of those players involved at the elite level of the game. Going forwards, primary and secondary preventative strategies for concussion injuries are a key priority for this setting, with the tackle event being the obvious point of focus for primary prevention efforts.

## Supplementary Information

Below is the link to the electronic supplementary material.

Supplementary file1 (DOCX 31 kb)

Supplementary file2 (PNG 228 kb)

## Declarations

### Availability of Data and Material (Data Transparency)

Open Science Framework at https://osf.io/z4xp7/files/

### Code Availability (Software Application or Custom Code)

https://osf.io/z4xp7/files/
