# Supplementary material for: Injuries in Elite Men’s Rugby Union: An Updated (2012–2020) Meta-Analysis of 11,620 Match and Training Injuries
Source: Sports Med. 2021 Dec 2;52(5):1127–40. doi: 10.1007/s40279-021-01603-w (PMC9023408; doi:10.1007/s40279-021-01603-w)
Supplement: Supplementary file 1 — Supplementary file1 (DOCX 31 kb) [file 40279_2021_1603_MOESM1_ESM.docx]

| **Reference** | **STROBE-SIIS Extension Item** | | | | | | | | | | | | | | | | | | | | | | | |
| --- | --- | --- | --- | --- | --- | --- | --- | --- | --- | --- | --- | --- | --- | --- | --- | --- | --- | --- | --- | --- | --- | --- | --- | --- |
|  | *1* | *2* | *3* | *4* | *5* | *6* | *7* | *8* | *9* | *10* | *11* | *12* | *13* | *14* | *15* | *16* | *17* | *18* | *19* | *20* | *21* | *22* | *23* | ***Total*** |
| Bitchell et al. ^[^[^19^](#_ENREF_19)^]^ | 1 | 1 | 0 | 1 | 1 | 1 | 1 | 1 | 0 | 0 | 1 | 0 | 0 | 1 | 1 | 1 | 1 | 1 | 1 | 1 | 0 | 1 | 1 | **17** |
| Cosgrave & Williams ^[^[^20^](#_ENREF_20)^]^ | 1 | 1 | 0 | 1 | 1 | 1 | 1 | 0 | 1 | 1 | 1 | 1 | 0 | 1 | 1 | 1 | 0 | 1 | 1 | 1 | 0 | 1 | 1 | **18** |
| Cousins et al. ^[^[^21^](#_ENREF_21)^]^ | 0 | 1 | 0 | 0 | 1 | 1 | 1 | 0 | 0 | 0 | 0 | 0 | 0 | 1 | 1 | 1 | 0 | 1 | 0 | 1 | 1 | 1 | 1 | **12** |
| Cruz-Ferreira et al. ^[^[^22^](#_ENREF_22)^]^ | 0 | 1 | 0 | 0 | 1 | 1 | 1 | 0 | 0 | 0 | 1 | 0 | 0 | 1 | 1 | 1 | 0 | 1 | 0 | 1 | 1 | 0 | 1 | **12** |
| Fuller et al. ^[^[^23^](#_ENREF_23)^]^ | 0 | 1 | 0 | 1 | 1 | 1 | 1 | 1 | 1 | 1 | 1 | 1 | 1 | 0 | 1 | 1 | 1 | 1 | 0 | 1 | 1 | 1 | 1 | **19** |
| Fuller et al. ^[^[^14^](#_ENREF_14)^]^ | 1 | 1 | 0 | 1 | 1 | 1 | 1 | 1 | 1 | 1 | 1 | 1 | 1 | 1 | 1 | 1 | 1 | 1 | 0 | 1 | 1 | 1 | 1 | **21** |
| Fuller et al. ^[^[^24^](#_ENREF_24)^]^ | 1 | 1 | 0 | 1 | 1 | 1 | 1 | 1 | 1 | 1 | 1 | 1 | 1 | 1 | 1 | 1 | 1 | 1 | 0 | 1 | 1 | 1 | 1 | **21** |
| Kemp et al. ^[^[^25^](#_ENREF_25)^]^ | 0 | 1 | 0 | 1 | 1 | 1 | 1 | 1 | 1 | 1 | 1 | 1 | 1 | 1 | 0 | 1 | 1 | 1 | 0 | 1 | 0 | 0 | 1 | **17** |
| Lanzetti et al. ^[^[^26^](#_ENREF_26)^]^ | 0 | 1 | 0 | 0 | 1 | 1 | 1 | 0 | 0 | 0 | 0 | 0 | 0 | 0 | 0 | 1 | 0 | 1 | 1 | 1 | 0 | 0 | 1 | **9** |
| Moore et al. ^[^[^27^](#_ENREF_27)^]^ | 0 | 1 | 0 | 1 | 1 | 1 | 1 | 1 | 1 | 1 | 1 | 1 | 1 | 0 | 1 | 1 | 1 | 1 | 0 | 1 | 1 | 1 | 1 | **19** |
| Rafferty et al. ^[^[^28^](#_ENREF_28)^]^ | 0 | 1 | 0 | 1 | 1 | 1 | 1 | 0 | 1 | 1 | 1 | 1 | 1 | 1 | 1 | 1 | 1 | 1 | 1 | 1 | 1 | 1 | 1 | **20** |
| Schwellnus et al. ^[^[^29^](#_ENREF_29)^]^ | 1 | 1 | 0 | 0 | 1 | 1 | 1 | 1 | 0 | 1 | 1 | 1 | 1 | 1 | 1 | 1 | 1 | 1 | 1 | 1 | 1 | 1 | 1 | **20** |
| Starling et al. ^[^[^30^](#_ENREF_30)^]^ | 0 | 1 | 0 | 1 | 1 | 1 | 1 | 0 | 0 | 0 | 1 | 1 | 1 | 1 | 1 | 1 | 1 | 1 | 1 | 1 | 0 | 0 | 0 | **15** |
| Starling et al. ^[^[^31^](#_ENREF_31)^]^ | 0 | 1 | 0 | 1 | 1 | 1 | 1 | 0 | 0 | 0 | 1 | 1 | 1 | 1 | 1 | 1 | 1 | 1 | 1 | 1 | 0 | 0 | 0 | **15** |
| Starling et al. ^[^[^32^](#_ENREF_32)^]^ | 0 | 1 | 0 | 1 | 1 | 1 | 1 | 0 | 0 | 0 | 1 | 1 | 1 | 1 | 1 | 1 | 1 | 1 | 1 | 1 | 0 | 0 | 0 | **15** |
| Stokes et al. ^[^[^7^](#_ENREF_7)^]^ | 1 | 1 | 0 | 1 | 1 | 0 | 1 | 0 | 0 | 0 | 0 | 1 | 1 | 1 | 1 | 1 | 1 | 1 | 0 | 1 | 1 | 1 | 0 | **15** |
| West et al. ^[^[^15^](#_ENREF_15)^]^ | 1 | 1 | 0 | 0 | 1 | 1 | 1 | 1 | 1 | 1 | 0 | 1 | 0 | 1 | 1 | 1 | 1 | 1 | 1 | 1 | 1 | 1 | 1 | **19** |
| West et al. ^[^[^8^](#_ENREF_8)^]^ | 0 | 1 | 0 | 0 | 1 | 1 | 1 | 1 | 0 | 1 | 0 | 1 | 0 | 1 | 1 | 1 | 1 | 1 | 1 | 1 | 1 | 1 | 1 | **17** |
| Whitehouse et al. ^[^[^33^](#_ENREF_33)^]^ | 0 | 1 | 0 | 1 | 1 | 1 | 1 | 1 | 1 | 1 | 1 | 1 | 1 | 1 | 0 | 1 | 1 | 1 | 0 | 1 | 1 | 0 | 1 | **18** |
|  |  |  |  |  |  |  |  |  |  |  |  |  |  |  |  |  |  |  |  |  |  |  | **Mean** | **17** |
|  |  |  |  |  |  |  |  |  |  |  |  |  |  |  |  |  |  |  |  |  |  |  | **SD** | **3** |
