# Supplementary figures and images for: Injuries in Elite Men’s Rugby Union: An Updated (2012–2020) Meta-Analysis of 11,620 Match and Training Injuries
Source: Sports Med. 2021 Dec 2;52(5):1127–40. doi: 10.1007/s40279-021-01603-w (PMC9023408; doi:10.1007/s40279-021-01603-w)

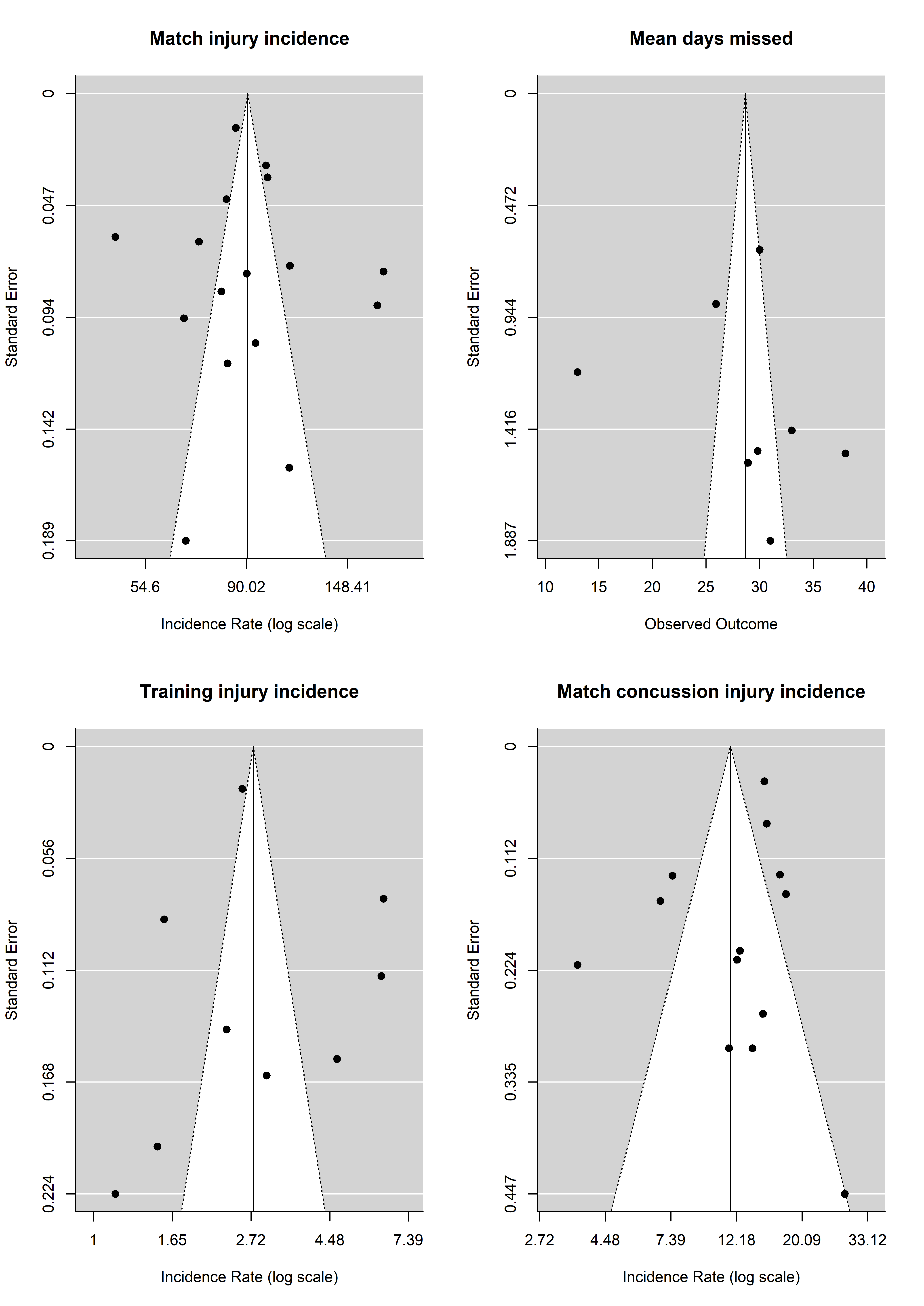

Supplement: Supplementary file 2 — Supplementary file2 (PNG 228 kb) [file 40279_2021_1603_MOESM2_ESM.png]
